# Occurrence of Antibiotic-Resistant Bacteria in Fish and Seafood from Slovak Market

**DOI:** 10.3390/foods12213912

**Published:** 2023-10-25

**Authors:** Monika Krahulcová, Klára Cverenkárová, Júlia Koreneková, Andrea Oravcová, Jana Koščová, Lucia Bírošová

**Affiliations:** 1 Faculty of Chemical and Food Technology, Department of Nutrition and Food Quality Assessment, Slovak University of Technology, Radlinského 9, 81237 Bratislava, Slovakia; klara.cverenkarova@stuba.sk (K.C.); julia.korenekova@stuba.sk (J.K.); andrea.oravcova@stuba.sk (A.O.); lucia.birosova@stuba.sk (L.B.); 2 Department of Microbiology and Immunology, University of Veterinary Medicine and Pharmacy in Košice, Komenského 73, 04181 Košice, Slovakia; jana.koscova@uvlf.sk

**Keywords:** microbiological analysis, antibiotic resistance, RTE food, sushi, poke, fish, seafood

## Abstract

The consumption of sushi or poke has grown globally. However, this type of dish often contains raw fish or seafood; therefore, it can pose a microbial risk for consumers. This study deals with the occurrence of total and antibiotic-resistant coliform bacteria and enterococci in fish and seafood as well as sushi and poke bought from Slovak retail (restaurants and fast food). Total coliforms have ranged in sushi, poke samples and samples of fish and seafood from cooling counters from 0.6 to 5.1 log CFU/g. Ampicillin resistance has been predominantly observed in all types of samples. Tetracycline resistance was detected in 16% of all tested samples and gentamicin resistance in 13%. Total enterococci has been detected in 74% of sushi samples, 100% of poke samples and 62% of samples obtained from supermarkets. The majority of enterococci were resistant to ampicillin. Vancomycin resistance was observed in five samples. Forty-eight resistant coliforms were identified mainly as *Enterobacter* spp. and *Klebsiella* spp. Antibiotic-resistant isolates were predominantly resistant to gentamicin, chloramphenicol and tetracycline. In 13% of resistant isolates was detected efflux pumps overproduction, and in four isolates was detected the *tetA* resistance gene. Our results point to poor hygiene in some establishments. The prevention of the antibiotic-resistant bacteria spread would be in better stewardship and improved monitoring of sanitation.

## 1. Introduction

Nowadays, consumers’ interest in food composition has increased significantly. Consumers are becoming more informed and critical in the context of food diet, for example by checking the fat content of food, due to its effects on the human cardiovascular system [1]. Seafood is considered a good source of protein, heart-healthy omega-3 fatty acids, vitamin D, selenium and iodine [2]. On the other hand, from the microbiological point of view, raw fish and seafood represent a good substrate for the growth of different bacteria. This is caused mainly by the increased post-mortem pH values. The contamination of fish meat can occur during further processing steps such as filleting or cutting through to the cross contamination of equipment and raw material or cooking personnel [3]. The globalization of the food market leads to the large-scale distribution of food, the increased movement of people and the forming of new consumer habits, which remain important attributes. To solve these problems, cooperation across different sectors is necessary with the same goal of preventing food-borne diseases and building an effective food safety system: the so-called “One Health approach” [4].

The current market offers fish and seafood in various forms, raw or processed. Sushi and poke containing raw seafood pose a risk for consumers’ health due to potential microbial contamination. Sushi is usually prepared as raw fish surrounded by cooked rice, all covered with algae [5]. Poke is served in bowls containing rice and generally raw fish cubed or sliced. Poke’s origin is Hawaii [6]. The fish in such a food is consumed in its raw form without previous thermal processing, and therefore, foodborne epidemics are relatively frequent. The most frequently detected bacterial contaminants are *Vibrio parahaemolyticus*, *Staphylococcus aureus* and various *Salmonella* species [7]. For example, in October 2021, a *Salmonella* spp. outbreak was recorded that was linked to the consumption of raw and cooked seafood which resulted with 115 illnesses in the USA [8]. Another salmonellosis outbreak has been reported due to the consumption of raw fish, sushi or poke in December 2022 in the USA, and the causative agent was raw salmon [9]. In a study conducted by Ramires et al. (2020), *Escherichia coli* O157:H7 possessing the virulence genes *eae*, *hlyA* and *stx1*, *stx2* was detected to be present in maki salmon sushi samples from restaurants in Brazil [10]. Fish and seafood produced in the aquatic industry are considered a potential source of shiga toxin-producing *E. coli* (STEC) [11]. *Staphylococcus aureus* is one of the most common causes of seafood-borne diseases worldwide, which are attributable to the contamination of food by preformed enterotoxins, especially SEA and SEB. Moreover, these strains are often *mec-A*-positive [12]. Another food-borne pathogen that is associated with illness in humans is *Listeria monocytogenes.* One of the main food vehicles for these bacteria is seafood products, including sushi, ceviche and poke [13].

The consumption of bacteria-contaminated food has caused more than half a billion cases of illness every year, while half a million cases end in death. The most susceptible groups are children younger than 5 years and old people. One of the factors contributing to mortality is the constant spread of antibiotic resistance among bacteria. Antibiotic resistance has been observed not only in clinical isolates but also in isolates of animal origin [4]. Currently, we are registering a significant threat of antibiotic resistance dissemination through the food chain [14]. Food may represent a good vector of antibiotic-resistant bacteria (ARB) and antibiotic resistance genes (ARGs) to consumers [15]. The current development of antimicrobial resistance in food pathogens is a matter of concern, in particular: fluoroquinolone resistance in *Enterobacterales*, the occurrence of methicillin-resistant *S. aureus* strains in animals and the worldwide occurrence of human and animal isolates of *E. coli* and *Salmonella* spp. producing extended-spectrum beta-lactamases [16].

According to the increasing problem of antibiotic resistance, we were interested in the occurrence of antibiotic-resistant indicator bacteria in selected food, which is gaining popularity among Slovak consumers. Indicator bacteria can point to insufficient hygiene, and if they are resistant, they can contribute to the spread of resistance. Therefore, we have aimed at the prevalence of antibiotic-resistant coliforms and enterococci in sushi and poke containing raw fish and seafood from different restaurants and supermarkets in Slovakia. Antibiotic-resistant isolates have been identified and characterized according to their susceptibility profile, their mechanism of antibiotic resistance and the presence of selected resistance genes.

## 2. Materials and Methods

### 2.1. Sampling

This study was performed using 50 samples of sushi collected from 11 sushi restaurants and 5 samples of poke collected from 3 restaurants in the capital of Slovakia, Bratislava. A sushi and poke were served for direct consumption. In the case of sushi, the analyzed part of the food was predominantly salmon (*n* = 21) and tuna (*n* = 14), supplemented by shrimps (*n* = 8), cuttlefish (*n* = 4), Japanese eel (*n* = 1), crab sticks (*n* = 1) and escolar (*n* = 1). In the case of poke, salmon (*n* = 3) and tuna (*n* = 2) were analyzed. Additionally, samples of cooled fishes and seafood sold on cooling counters that were determined to not be only for direct consumption were tested. Cooled fishes and seafood were gathered from three different supermarkets. Thirteen samples of cooled fishes and seafood were analyzed, specifically halibut (*n* = 5), salmon (*n* = 4), shrimp (*n* = 3) and tuna (*n* = 1). The samples were taken in summer season of the year (June–August). Immediately after purchase, the samples were conducted for microbiological analyses (within 3 h). The summarization of the analyzed samples is listed in Table 1.

### 2.2. Monitoring of Total and Antibiotic-Resistant Bacteria Occurrence

The samples of fish and seafood were weighed and homogenized in a sterile saline solution in a ten-fold dilution using a Stomacher device (VWR, Leuven, Belgium). A volume of 100 μL was aseptically inoculated onto the solidified culture medium. Monitoring of total and antibiotic-resistant bacteria was performed by diagnostic media with and without antibiotics (ATBs) in three parallels. Agar plates with ATBs were prepared by adding the selected ATBs into the slightly cooled medium before being well mixed to ensure its efficiency. Coliform bacteria were detected with Chromocult coliform agar (VWR, Darmstadt, Germany). Antibiotic-resistant coliform bacteria were detected with the use of ampicillin, gentamicin, ciprofloxacin, chloramphenicol and tetracycline of different concentration chosen according to the resistance breakpoints given by the European Committee on Antimicrobial Susceptibility Testing (EUCAST) for *Enterobacterales* [17]. The concentration of antibiotic tetracycline was chosen according to the Clinical & Laboratory Standards Institute (CLSI) [18]. European guidelines do not list tetracycline for *Enterobacterales*. Enterococci were detected with Slanetz–Bartley agar (Biolife Italiana srl.). Antibiotic-resistant enterococci were detected with the use of ampicillin, gentamicin, ciprofloxacin and vancomycin of different concentration according to European guidelines [17]. Antibiotics were added into cooled medias (Chromocult Coliform agar, Slanetz–Bartley), well mixed and poured into plates under aseptic conditions. Plates used to monitor coliform bacteria were incubated at 37 °C for 24 h and enterococci were incubated at 40 °C for 48 h. The enumerations of total coliform bacteria (CFB), enterococci (ENC) and ARB were statically evaluated using Student’s *t*-test.

### 2.3. Identification of Antibiotic-Resistant Bacteria Isolates

Antibiotic-resistant isolates were randomly selected and inoculated on Mueller–Hinton agar medium (BioLife, Monza, Italy) to obtain separate colonies. Agar plates were incubated (37 °C, 24 h) and the grown colonies were identified using matrix-assisted laser desorption/ionization–time of flight (MALDI-TOF) mass spectrometry analysis. Separated grown colonies of resistant bacteria was spotted onto a steel target plate (Bruker Daltonics Inc., Billerica, MA, USA) and covered by 1 μL of matrix solution. The matrix solution of α-cyano-4-hydroxycinnamic acid was prepared as a saturated solution in 50% acetonitrile and 2.5% trifluoroacetic acid. Consequently, the steel target plate was left to dry at room temperature. Samples were evaluated by the use of an AutoFlex I TOF-TOF apparatus (Bruker Daltonics Inc., Billerica, MA, USA) in linear positive-ion mode across the *m*/*z* range from 2000 to 20,000, with the gating of ions below *m*/*z* 400 and a delayed extraction time of 450 ns. Each spot-on target steel plate was measured with 2000 laser shots at 25 Hz in groups of 50 shots per sampling area of the spot. The data sampling rate was 0.5 GHz. Each plate was calibrated by using a bacterial test standard (Bruker Daltonics). Spectra were analyzed by using MALDI BioTyper software (v 2.0) (BioTyper Library v 3.0; Bruker Daltonics) for spectral pattern in a logarithmic score from 0 to 3, where a score of >1.9 indicates species identification, a score from 1.7 to 1.9 indicates genus identification and a score of <1.7 indicates no identification [19].

### 2.4. Detection of Sensitivity Profile in Antibiotic-Resistant Coliform Bacteria Isolates

The detection of a sensitivity profile of antibiotic-resistant isolates was performed using the macro-dilution agar drop method with ATBs: ciprofloxacin, gentamicin, ampicillin, tetracycline, chloramphenicol, ceftazidime and meropenem. Identified resistant colonies were incubated overnight in Mueller–Hinton broth (BioLife, Italy) (37 °C, 16 h). Overnight cultures of isolates were diluted to 0.5 McFarland and pipetted onto agar plates of Mueller–Hinton agar supplemented with ATBs. The concentration of ATBs was defined by resistant breakpoints given by EUCAST and CLSI. The growth of colonies on agar plates were visually evaluated after 24 h at 37 °C [19]. The experiments were repeated three times.

### 2.5. Detection of Overproduction of Efflux Pumps in Antibiotic-Resistant Coliform Bacteria Isolates

Detection of efflux pumps in antibiotic-resistant coliform bacteria isolates was performed via the EtBr agar Carthweel method [20]. The evaluation of the experiment was visual through UV irradiation, due to EtBr being a fluorescent active compound (Serva, Germany). The over-production of efflux pumps was detected according to Lépesová et al. (2020) [21]. The reference strain of *E. coli* (CCM 3988) from the collection of Czech Collection of Bacterial Strains in Brno was used as negative control. The experiments were repeated three times and ran in triplicates.

### 2.6. Detection of Resistance Genes in Antibiotic-Resistant Coliform Bacteria Isolates

The detection of resistance genes in antibiotic-resistant coliform bacteria isolates was performed via multiplex and single polymerase-chain reaction (PCR). Evaluation of sensitivity profile of isolates determine selected resistance genes. Resistance genes detected in this study were beta-lactamases *bla*_TEM_, *bla*_SHV_, *bla*_OXA_ and *bla*_CTX-M_ (Group 1) [22] and tetracycline resistance genes Group 2: *tetA*, *tetE* [23]. PCR conditions, reagents and primers were prepared according to Lépesová et al. (2020) [21]. Each reaction had a negative and positive control. The positive controls used during reactions were sequenced for the presence of specific resistance genes. The evaluation of PCR products was conducted through gel electrophoresis, with the use of 1.5% agarose dissolved in TAE buffer. Electrophoresis was set up for 1 h and 45 min at 100 V and the gel was additionally stained with Gel Red (Biotium, CA, USA) during the next 30 min. The PCR products were visualized under UV irradiation.

## 3. Results and Discussion

Approximately 90% of aquaculture production is located in developing countries with poor hygiene standards and incomplete regulations specifying the use of ATBs. Antimicrobial substances are often used to control diseases, minimize economic loss or speed up production to cover the growing demand. In such systems, ARGs are repeatedly transferred into the gut microbiota of livestock intended for human consumption [24]. Nevertheless, information on the occurrence of ARB and ARGs in these fish and seafood is still limited [5,25].

A total of 50 sushi samples were analyzed (Table 2). 

A more detailed description of the tested samples is presented in Table 1 in the Materials and Methods section. The bacteria *E. coli* as well as its resistant variants were not detected in any type of tested sushi samples. The number of total CFB have ranged from 0.6 to 4.3 log CFU/g, while in 10% of sushi samples CFB were not observed (Table 2). The increased numbers of CFB could be caused by the period of purchasing the samples, as certain differences are described depending on the winter and summer samplings. The samples analyzed in this study were purchased during summer season. A higher occurrence of the monitored bacteria is reported in the summer months [25]. Ramires et al. (2020) also detected CFB in sushi samples with salmon in different establishments. Coliform bacteria were found in each of the monitored samples, with an average number of 2.34 log CFU/g [10].

Antibiotic-resistant CFB were observed on diagnostic media supplemented with ATBs in EUCAST resistance breakpoints concentration. We have chosen ATBs from different antibiotic classes. Ampicillin-resistant CFB were present in 84% of detected sushi samples (Table 2). Tetracycline resistance was present in 20% of all sushi samples.

The presence of CFB as well as antibiotic-resistant CFB were specifically linked to food establishment. The most significant differences were observed in food establishments E and F (*p* < 0.05). The results indicate that the contamination is mainly from the environment of food service establishment or the manipulating personnel. It should not be forgotten that sushi is a food commodity prepared exclusively by the cook’s hands (wrapping in rice and seaweed) and since most types of sushi do not undergo additional heat treatment, the probability of contamination increases. Therefore, the microbiological analysis of sushi products does not only reflect the microbial aspect of the raw materials used to prepare the food (fish, rice, seaweed), but also the hygiene of the personnel who prepared the food [5,26,27]. Atanassova et al. (2008) investigated the difference between frozen sushi obtained in supermarkets and freshly prepared sushi in restaurants. Also, they proved that the microbial quality of sushi depends exclusively on the skills and hygienic habits of the staff preparing it [28]. The same results were reached by Batista et al. (2017), who monitored the occurrence of CFB in sushi samples in restaurants and they also swabbed the hands of the cooking personnel. They clearly demonstrated that workers handling ready-to-eat foods significantly affected the microbial quality of the final product [29].

Enterococci were recorded in 74% of sushi samples and ranged from 0.1 to 4.2 log CFU/g (Table 2). The most significant differences were observed in food establishments E, F, J and K (*p* < 0.05). Gentamicin and ciprofloxacin resistance were not detected. Ampicillin resistance was dominant and was observed in 34% of samples. Vancomycin resistance was observed in two samples of sushi. Vancomycin is a glycopeptide ATB, which binds to the terminal part of peptidoglycan precursors and inhibits cross-linking of the cell wall. Nine vancomycin resistance genes in enterococci (*vanA*, *vanB*, *vanC*, *vanD*, *vanE*, *vanG*, *vanL*, *vanM*, *vanN*) have been described. In general, these genes encode enzymes synthesizing new peptidoglycan precursors and enzymes destroying d-Ala-d-Ala-ending precursors. One of the biggest problems is spreading these resistance genes into more pathogenic microorganisms such as methicillin-resistant *Staphylococcus aureus* [30]. However, there is still a lack of information about antibiotic-resistant ENC occurrence in fish, despite its diversity and frequent occurrence in such a food. The most important method of contamination could be during dissection where ENC from the gut could be transferred to fish meat [29,31].

Microbiological criteria for ready-to-eat products, including the tested sushi food, are defined by the European regulation EC Reg. 2073/2005, which applies two criteria. Specifically, it is the absence of *Salmonella* spp. in 25 g of sample and the maximum presence of the bacterium *Listeria monocytogenes* in the number of 100 CFU/g [32]. Limits are not set for the presence of ENC, CFB or *E. coli*, even though their presence often indicates the possible occurrence of pathogenic microorganisms.

Since poke dishes are very similar to sushi, and poke has gained popularity in Slovakia as well, total and antibiotic-resistant CFB (including *E. coli*) and ENC were detected in poke samples. There were no significant differences between different food establishments in the case of CFB and ENC (*p* < 0.05). Five samples of fishes originating from dish poke were tested (three samples of salmon and two samples of tuna). The total CFB detected in each sample ranged from 3.4 to 4.5 log CFU/g (Table 3). These counts are a little bit higher compared to the study conducted by Marquis et al. (2023) who detected a lower number of total CFB (2.29 log CFU/g) in California, USA [33]. The majority of CFB has shown ampicillin and gentamicin resistance. Tetracycline resistance was observed in only one poke sample. Ciprofloxacin and chloramphenicol resistance were not detected. None of the poke samples contained *E. coli*. Total ENC were detected in all tested poke samples in numbers from 2.0 to 4.4 log CFU/g. All assessed samples contained ampicillin-resistant ENC in the range from 2.0 to 4.2 log CFU/g. Ampicillin is a representative agent of group β-lactam ATBs as it inhibits the synthesis of the cell wall component peptidoglycan in enterococci. Intrinsic tolerance against ampicillin is associated with a species-specific enterococci chromosomal gene encoding enzymes with a low binding affinity for β-lactams. Another form of ampicillin resistance could be through the destruction of the β-lactam ring by β-lactamases, originally described in genus staphylococci or by developing transpeptidases, which is more likely involved in the maintenance of peptidoglycan [30,34]. Gentamicin, ciprofloxacin and vancomycin resistance were not detected. Poke bowls are becoming more popular, as it represents a combination of the health benefits of fish, salad and vegetables, complemented by a tasty sauce [35]. However, this dish contains raw fish, which may still represent microbiological risk for the consumer due to presence of pathogenic or potential pathogenic bacteria.

In addition, samples of cooled fishes and seafood sold through cooling counters were analyzed. These cooled fishes and seafood are not intended for only direct consumption. On the other hand, some consumers use them to prepare homemade tartare from raw fish, which is gaining on popularity. From this point of view, the microbiological risk of cooled fish for these consumers should be taken into account. Total CFB has been detected in the range from 2.2 to 5.1 log CFU/g in the samples of cooled fishes and seafood (Table 4). There were no significant differences between different food entablement in the case of CFB (*p* < 0.05).

Ampicillin- and gentamicin-resistant CFB have been observed, with ampicillin resistance appearing in 85% of the cooled fish samples originating from different markets. Gentamicin-resistant CFB have been detected in only one sample of salmon. The bacterium *E. coli* was detected in approximately half of the cooled fishes and seafood samples in the number from 2.0 to 3.3 log CFU/g (Table 4). Antibiotic-resistant *E. coli* were only found in the sample of cooled halibut. The presence of *E. coli* in cooled fish from supermarkets and fish markets was also observed in other countries with a higher consumption of fish. Moreover, the *E. coli* O157:H7 serotype has been also detected in ready-to-eat sushi in Brazil, 2019 [10]. In Portugal, *E. coli* was found in tuna and salmon [26]. Total ENC were detected in 62% of the samples and their number ranged from 3.1 to 4.2 log CFU/g (Table 4). The most significant differences were observed in food establishment 3 (*p* < 0.05). Only vancomycin resistance was observed in three samples obtained from same food establishment. Ampicillin, gentamicin and ciprofloxacin resistance was not detected. Vancomycin-resistant enterococci were also determined in even half samples of cooled fishes distributed in commercial markets in Japan [29]. Enterococci were also detected in tuna and shrimp samples from supermarkets in Switzerland [24].

### 3.1. Antibiotic-Resistant Isolates Identification and Characterization of Their Antibiotic Susceptibility Profile

Sixty antibiotic-resistant strains were successfully identified. Twelve of them were identified as enterococci, specifically *Enterococcus casseliflavus* (*n* = 5), *E. gallinarum* (*n* = 3), *E. gilvus* (*n* = 3) and *E. faecium* (*n* = 1). Most of the identified antibiotic-resistant enterococci were isolated from salmon (58%) and tuna (22%) samples, which were also the most tested fish samples according to consumer preferences. The rest of the species were isolated from shrimp and halibut. Enterococcal species had very weak viability and did not survive further processing. Therefore, enterococci were not further characterized.

**Figure 1:**
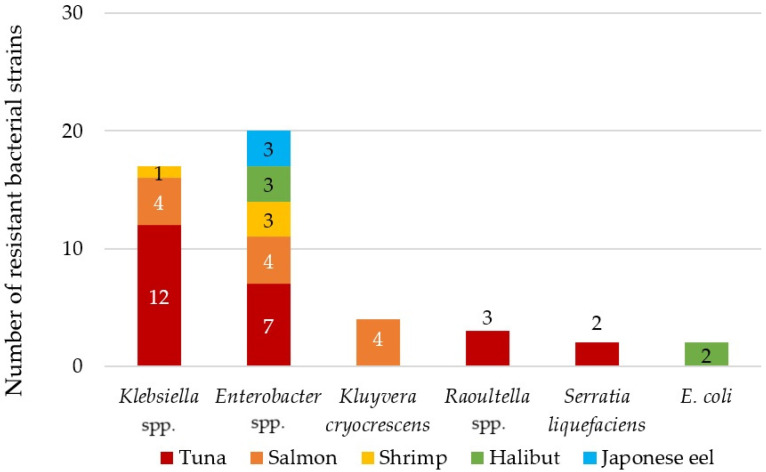
Distribution of identified antibiotic-resistant bacteria isolated from tested sushi, cooled fishes and seafoods and poke samples.

Forty-eight antibiotic-resistant CFB were isolated from samples of sushi, poke, cooled fish and seafood in Slovak retail shops. The majority were identified as *Enterobacter* spp. (42%) and *Klebsiella* spp. (35%) (Figure 1). Antibiotic-resistant *E. coli* was isolated only from a sample of cooled halibut from supermarkets. The remaining ARB were identified as *Kluyvera cryocrescens* (8%), *Raoultella* spp. (6%) and *Serratia liquefaciens* (4%) (Figure 1). Most of the identified antibiotic-resistant strains were isolated from tuna (50%) and salmon (25%) samples, which were also the most tested fish samples according to consumer preferences. Bacteria of the genus *Enterobacter* spp. were also dominant in the sushi samples in the study by Vitas et al. (2018) in Spain [36]. Strains of the genera *Klebsiella* spp., *Serratia* spp., *Kluyvera* spp. and *Raoultella* spp. were previously identified in fish samples from sushi dishes as well [5,37], as in our case (Figure 1).

The antibiotic susceptibility profile of the isolates was determined using different ATBs. All resistant isolates were ampicillin resistant (Table 5). Ampicillin resistance is intrinsic in majority of CFB. The only exception is *E. coli*, which is supposed to be sensitive to beta-lactam ATBs [38]. 

Resistance to gentamicin and chloramphenicol was observed in 54% and 35%, respectively. Gentamicin resistance was predominantly detected in *Klebsiella* spp. (50%) and *Enterobacter* spp. (38%), as the rest of the resistant isolates were of the genus *Raoultella* spp. The resistance to chloramphenicol was observed in *Enterobacter* spp. (59%), *Klebsiella* spp. (29%) and *S. liquefaciens* (12%) and isolated from fish and seafood from supermarket cooling counters and sushi. This type of antibiotic resistance used to be detected in bacteria isolated from dishes such as sushi [36,39].

Tetracycline resistance was observed in 8% of the CFB antibiotic-resistant strains identified as *K. cryocrescens* isolated from cooled salmon and sushi samples. Whereas there are no tetracycline resistance breakpoints for *Enterobacterales* in EUCAST guidelines, CLSI breakpoints were applied. Clinical bacteremias caused by *K. cryocrescens* are often treated by the administration of the ATB class of tetracyclines, but infections caused by this agent are very rare [40]. Bacteria of the genus *Kluyvera* spp. are commonly considered as environmental strains, but it should be noted that it may be a potential reservoir for the spread of ARGs, as, e.g., the strain *K. cryocrescens* has a chromosomally bound beta-lactamase that is 85% identical to the ESBL resistance gene CTX-M-1 [41,42]. Currently, novel ESBLs variants of CTX-M genes, namely CTX-M-152, which is a part of *bla*_CTX-M-25_ group detected in *Kluyvera* sp. species were identified in the environmental wastewater samples acquired in India [43].

Ciprofloxacin, ceftazidime and meropenem resistance were not observed in any antibiotic-resistant isolates.

Bacteria did not exhibit multidrug resistance to three and more ATBs belonging to different antibiotic classes [44]. None of the isolated antibiotic-resistant strains were multidrug resistant.

### 3.2. Identification of Selected Resistance Mechanisms in Antibiotic-Resistant Isolates

Efflux pumps are naturally occurring in Gram-positive and Gram-negative bacterial cells and serve as transmembrane proteins. The overproduction of efflux pumps is considered as one of the most important resistance mechanisms, as they may exclude ATBs from intracellular space. In MDR bacteria, the overproduction of efflux pumps is a common resistance mechanism as they do not need to be ATB specific, and one type of efflux pump may exclude various ATBs [45].The overproduction of efflux pumps was detected in only 13% of resistant isolates, especially *K. cryocrescens* (*n* = 4) and *E. cloacae* (*n* = 2) (Table 5).

Based on the antibiotic-sensitivity profile of bacterial isolates selected ARGs were chosen, which were consequently detected with end-point PCR. Bacterial isolates originating from sushi, poke, fish and seafood distributed on cooling counters in supermarkets had been predominantly detected with gentamycin, chloramphenicol and tetracycline resistance. From all studied ARGs, only the *tetA* resistance gene was detected in the isolates presented in this study. All tetracycline-resistant *K. cryocrescens* were detected with the *tetA* resistance gene (Table 5). Tetracycline-resistant genes *tetA* and *tetE* are encoding active efflux pumps [46]. In the isolates of *K. cryocrescens* in the presented study, the over-production of efflux pumps was detected. Another gene-encoding efflux pump, *tetE*, was not detected. Resistance genes encoding tetracycline resistance were detected in the environmental isolates of *Kluyvera* sp. before. For example, in water samples obtained in Egypt *tetA* genes in most of the isolates of *Kluyvera* sp. were detected [47]. Also, seawater samples with *tetA* resistance genes in China were predominantly detected [46]. Tetracycline is an ATB frequently applied in veterinary medicine, as are chlortetracycline, oxytetracycline and doxycycline. In addition to therapeutic purposes, in many countries, tetracyclines are often incorporated into livestock feed at subtherapeutic doses as growth promoters for swine, poultry and even for aquaculture [48]. It is hard to evaluate the possible impact of the numerous prescriptions of ATBs being released into the environment. The truth is that the quantity of ATBs applied in human medicine is lower than in veterinary care [49]. Moreover, the tetracycline class of ATBs is hardly metabolized in human or animal bodies and non-metabolized forms enter the environment through urea or feces [50]. These substances create a pressure on the microbial community in the environment and end in the selective forming of ARB and ARGs [51].

## 4. Conclusions

Seafood and fish have gained popularity among consumers in recent years. Moreover, the aquaculture environment creates the perfect conditions for the development and constant spread of ARB and it serves as a reservoir of ARGs. Fish and shellfish raised in crowded and stressful environments are more susceptible to bacterial infections, which increases the prophylactic and therapeutic use of antimicrobial agents, such as ATBs. The findings of this study have shown that fish and seafood distributed by restaurants and supermarkets intended for direct or undirect consuming, in the forms of sushi, poke or cooling counters contain ARB. There were 48 resistant coliform bacteria identified, mainly of the genera *Enterobacter* spp. and *Klebsiella* spp., but also of *E. coli*. Antibiotic-resistant isolates were predominantly resistant to gentamicin, chloramphenicol and tetracycline. Only 13% of resistant isolates were evaluated with the overproduction of efflux pumps, and in four cases of isolates the *tetA* resistance gene was detected. The results of this study confirm the need for the One Health approach and cooperation across different sectors as contamination from food-borne diseases may rise from the environment, materials, cooking personnel and many other factors. Our data also showed that the occurrence of antibiotic-resistant bacteria is more likely connected to FSE than to the food matrix. Therefore, the prevention of the antibiotic-resistant bacteria spread would be in better stewardship and the improved monitoring of sanitation.

## Figures and Tables

**Table 1:** Details of tested samples of fish and seafood from sushi, poke and supermarkets sold on cooling counters.

Sushi Samples		
FSE A	FSE E	FSE I
M: Salmon	M: Salmon	M: Crab sticks
M: Tuna	N: Salmon	M: Salmon
M: Shrimp	N: Tuna	N: Tuna
FSE B	FSE F	N: Tuna
M: Salmon	M: Tuna	N: Salmon
N: Shrimp	M: Salmon	N: Cuttlefish
N: Tuna	N: Shrimp	N: Shrimp
N: Salmon	N: Cuttlefish	N: Escolar
M: Tuna	N: Salmon	FSE J
FSE C	N: Tuna	M: Salmon
N: Tuna	FSE G	M: Salmon
N: Salmon	M: Salmon	N: Salmon
N: Shrimp	FSE H	N: Tuna
M: Salmon	N: Cuttlefish	FSE K
M: Salmon	N: Shrimp	N: Shrimp
FSE D	N: Salon	N: Tuna
N: Tuna	N: Tuna	N: Salmon
N: Salmon	M: Salmon	M: Tuna
N: Shrimp		M: Salmon
N: Japanese eel		
N: Cuttlefish		
Poke Samples		
**FSE E**	FSE G	FSE L
Salmon	Salmon	Salmon
	Tuna	Tuna
Cooled Fish Sample		
**Supermarket 1**	**Supermarket 2**	Supermarket 3
Shrimp	Halibut	Shrimp
Shrimp	Halibut	Tuna
Halibut	Salmon	Salmon
Halibut	Salmon	Salmon Keta
		Halibut

M—maki type of sushi, N—nigiri type of sushi, FSE—food service establishment.

**Table 2:** Total and antibiotic-resistant fecal indicator bacteria in sushi containing seafood.

FSE	AFS	VS	TCB	Antibiotic-Resistant Coliform Bacteria					TEN	Antibiotic-Resistant Enterococci	
				AMP	GEN	CIP	CHL	TET		AMP	VAN
A	SL	N	3.0 ± 0.15	2.9 ± 0.13	ND	ND	ND	ND	ND	ND	ND
	TN	N	1.1 ± 0.05	0.8 ± 0.09	ND	ND	ND	ND	ND	ND	ND
	SHR	N	1.3 ± 0.04	0.5 ± 0.02	0.1 ± 0.01	ND	ND	ND	ND	ND	ND
B	SL	M	1.9 ± 0.04	1.2 ± 0.08	ND	ND	ND	ND	1.1 ± 0.04	0.7 ± 0.02	ND
	SHR	N	1.5 ± 0.07	0.1 ± 0.02	ND	ND	ND	ND	0.1 ± 0.02	ND	ND
	TN	N	2.6 ± 0.09	2.6 ± 0.10	2.2 ± 0.09	ND	ND	ND	0.7 ± 0.04	0.4 ± 0.01	ND
	SL	N	1.7 ± 0.02	ND	ND	ND	ND	ND	1.0 ± 0.07	1.0 ± 0.08	ND
	TN	M	2.2 ± 0.11	1.6 ± 0.09	ND	ND	ND	ND	1.0 ± 0.05	0.3 ± 0.01	ND
C	TN	N	ND	ND	ND	ND	ND	ND	0.9 ± 0.08	0.5 ± 0.05	ND
	SL	N	ND	ND	ND	ND	ND	ND	0.8 ± 0.03	ND	ND
	SHR	N	ND	ND	ND	ND	ND	ND	0.5 ± 0.02	0.3 ± 0.01	ND
	SL	M	0.6 ± 0.01	ND	ND	ND	ND	ND	0.3 ± 0.02	ND	ND
	SL	M	0.7 ± 0.01	0.3 ± 0.02	ND	ND	ND	ND	1.2 ± 0.07	1.0 ± 0.09	ND
D	TN	N	2.2 ± 0.10	2.0 ± 0.07	ND	ND	ND	ND	1.5 ± 0.08	0.9 ± 0.07	ND
	SL	N	1.9 ± 0.12	1.8 ± 0.11	ND	ND	ND	ND	ND	ND	ND
	SHR	N	1.3 ± 0.09	1.2 ± 0.05	ND	ND	ND	ND	0.6 ± 0.04	0.5 ± 0.04	ND
D	JPE	N	1.2 ± 0.05	1.0 ± 0.06	ND	ND	ND	ND	ND	ND	ND
	CF	N	1.3 ± 0.04	1.2 ± 0.02	ND	ND	ND	ND	ND	ND	ND
E	SL	M	4.3 ± 0.24	4.1 ± 0.20	ND	ND	ND	3.5 ± 0.13	4.1 ± 0.17	4.0 ± 0.27	ND
	SL	N	4.1 ± 0.20	3.0 ± 0.19	ND	ND	ND	3.5 ± 0.16	4.2 ± 0.11	4.1 ± 0.30	ND
	TN	N	4.2 ± 0.20	4.2 ± 0.45	ND	ND	ND	3.6 ± 0.17	4.1 ± 0.20	4.1 ± 0.31	ND
F	TN	M	4.1 ± 0.19	2.2 ± 0.08	ND	ND	ND	3.6 ± 0.10	4.1 ± 0.07	ND	ND
	SL	M	4.1 ± 0.30	1.9 ± 0.08	ND	ND	ND	3.6 ± 0.19	4.1 ± 0.13	ND	ND
	SHR	N	4.1 ± 0.42	2.6 ± 0.15	ND	ND	ND	3.6 ± 0.17	4.1 ± 0.21	ND	ND
	CF	N	4.2 ± 0.25	2.6 ± 0.07	ND	ND	ND	3.7 ± 0.13	4.1 ± 0.20	2.0 ± 0.08	ND
	SL	N	4.2 ± 0.20	2.6 ± 0.11	ND	ND	ND	3.7 ± 0.20	4.1 ± 0.15	2.0 ± 0.07	ND
	TN	N	4.2 ± 0.31	2.6 ± 0.13	ND	ND	ND	3.7 ± 0.17	4.2 ± 0.08	2.0 ± 0.10	ND
G	SL	M	1.9 ± 0.12	1.9 ± 0.09	ND	ND	ND	ND	1.9 ± 0.05	ND	ND
H	CF	N	ND	ND	ND	ND	ND	ND	ND	ND	ND
	SHR	N	2.9 ± 0.30	2.6 ± 0.15	ND	ND	ND	ND	ND	ND	ND
	SL	N	ND	ND	ND	ND	ND	ND	ND	ND	ND
	TN	N	2.0 ± 0.36	2.0 ± 0.15	2.0 ± 0.12	ND	ND	ND	ND	ND	ND
	SL	M	2.0 ± 0.19	2.0 ± 0.17	ND	ND	ND	ND	ND	ND	ND
I	CTS	M	3.9 ± 0.55	3.5 ± 0.31	ND	ND	ND	2.0 ± 0.12	2.3 ± 0.09	ND	ND
	SL	M	2.2 ± 0.17	1.9 ± 0.08	ND	ND	ND	ND	2.2 ± 0.10	ND	ND
	TN	N	2.9 ± 0.40	2.2 ± 0.11	ND	ND	ND	ND	2.5 ± 0.07	ND	ND
	TN	N	3.1 ± 0.48	2.7 ± 0.20	ND	ND	ND	ND	2.9 ± 0.11	ND	ND
	SL	N	2.4 ± 0.21	2.2 ± 0.19	ND	ND	ND	ND	2.6 ± 0.07	ND	ND
	CF	N	2.7 ± 0.16	2.4 ± 0.21	ND	ND	ND	ND	ND	ND	ND
	SHR	N	3.3 ± 0.30	3.0 ± 0.29	ND	ND	ND	ND	ND	ND	ND
	EC	N	2.4 ± 0.15	ND	ND	ND	ND	ND	2.8 ± 0.12	ND	ND
J	SL	M	3.3 ± 0.33	3.1 ± 0.25	ND	ND	ND	ND	3.7 ± 0.27	ND	ND
	SL	M	3.2 ± 0.30	2.9 ± 0.17	ND	ND	ND	ND	3.2 ± 0.21	ND	ND
	SL	N	3.3 ± 0.28	2.8 ± 0.18	ND	ND	ND	ND	3.4 ± 0.25	ND	ND
	TN	N	3.5 ± 0.51	3.0 ± 0.22	ND	ND	2.1 ± 0.10	ND	3.5 ± 0.25	ND	ND
K	SHR	N	2.8 ± 0.25	1.4 ± 0.06	ND	2.7 ± 0.19	ND	ND	3.0 ± 0.18	ND	1.9 ± 0.08
	TN	N	3.0 ± 0.22	1.8 ± 0.08	ND	2.2 ± 0.20	ND	ND	3.2 ± 0.15	ND	ND
	SL	N	3.1 ± 0.29	1.8 ± 0.08	ND	1.9 ± 0.10	ND	ND	3.2 ± 0.20	ND	ND
	TN	M	2.8 ± 0.16	1.0 ± 0.04	ND	ND	ND	ND	3.3 ± 0.25	ND	ND
	SL	M	3.3 ± 0.28	1.7 ± 0.10	1.3 ± 0.11	2.9 ± 0.15	ND	ND	3.4 ± 0.27	ND	1.6 ± 0.05

FSE—food service establishment; AFS—analyzed fish or seafood, SL—salmon, TN—tuna, SHR—shrimp, CF—cuttlefish, JPE—Japanese eel, CTS—crab sticks, EC—escolar; VS—variant of sushi, N—nigiri, M—maki; TCB—total coliform bacteria; TEN—total enterococci; AMP—ampicillin; GEN—gentamycin; CIP—ciprofloxacin; CHL—chloramphenicol; TET—tetracycline; VAN—vancomycin; ND—not detected.

**Table 3:** Total and antibiotic-resistant fecal indicator bacteria in poke containing seafood.

FSE	AFS	TCB	Antibiotic-Resistant Coliform Bacteria			TEN	Antibiotic-Resistant Enterococci
			AMP	GEN	TET		AMP
E	SL	4.5 ± 0.39	4.2 ± 0.35	2.2 ± 0.16	2.69 ± 0.15	4.0 ± 0.22	3.8 ± 0.26
G	SL	3.5 ± 0.33	3.4 ± 0.19	2.3 ± 0.19	ND	2.0 ± 0.06	2.0 ± 0.09
	TN	3.4 ± 0.28	3.3 ± 0.14	ND	ND	4.4 ± 0.35	4.2 ± 0.31
L	SL	3.4 ± 0.25	3.2 ± 0.11	2.0 ± 0.10	ND	3.7 ± 0.21	3.5 ± 0.25
	TN	3.5 ± 0.20	3.4 ± 0.15	2.3 ± 0.12	ND	4.1 ± 0.37	3.9 ± 0.30

FSE—food service establishment; AFS—analyzed fish or seafood, SL—salmon, TN—tuna; TCB—total coliform bacteria; TEN—total enterococci; AMP—ampicillin; GEN—gentamycin; CIP—ciprofloxacin; CHL—chloramphenicol; TET—tetracycline; VAN—vancomycin; BDL—below the detection limit.

**Table 4:** Total and antibiotic-resistant fecal indicator bacteria in cooled fishes and seafood from supermarkets.

Supermarket	AFS	TCB	TEC	Antibiotic-Resistant Coliform Bacteria		TEN	Antibiotic-Resistant Enterococci
				AMP	GEN		VAN
1	SHR	4.1 ± 0.30	ND	4.0 ± 0.25	ND	4.0 ± 0.32	2.9 ± 0.17
	SHR	4.0 ± 0.28	ND	3.9 ± 0.29	ND	3.8 ± 0.26	ND
	HB	4.5 ± 0.28	ND	4.4 ± 0.34	ND	4.2 ± 0.37	3.1 ± 0.25
	HB	4.7 ± 0.33	2.0 ± 0.10	4.4 ± 0.27	ND	4.1 ± 0.35	2.2 ± 0.18
2	HB	5.1 ± 0.39	2.0 ± 0.09	5.1 ± 0.41	ND	3.1 ± 0.23	ND
	HB	5.1 ± 0.40	2.5 ± 0.12	5.1 ± 0.39	ND	3.2 ± 0.22	ND
	SL	3.7 ± 0.29	3.3 ± 0.23	3.3 ± 0.25	ND	3.9 ± 0.20	ND
	SL	3.6 ± 0.25	3.1 ± 0.20	3.5 ± 0.22	ND	3.9 ± 0.30	ND
3	SHR	2.2 ± 0.15	ND	ND	ND	ND	ND
	TN	2.2 ± 0.11	ND	ND	ND	ND	ND
	SL	2.6 ± 0.10	ND	2.0 ± 0.14	2.0 ± 0.21	ND	ND
	SL	3.7 ± 0.26	2.6 ± 0.19	3.2 ± 0.22	ND	ND	ND
	HB	3.5 ± 0.30	ND	3.4 ± 0.15	ND	ND	ND

FSE—food service establishment; AFS—analyzed fish or seafood, SHR—shrimp, HB—halibut, SL—salmon, TN—tuna; TCB—total coliform bacteria; TEN—total enterococci; AMP—ampicillin; GEN—gentamycin; CIP—ciprofloxacin; CHL—chloramphenicol; TET—tetracycline; VAN—vancomycin; ND—not detected.

**Table 5:** Antibiotic susceptibility profile, production of efflux pumps and detected resistance genes of antibiotic-resistant isolates.

Sample	Resistant Isolate	Antibiotic Susceptibility Profile							O-EP	RG	Sample	Resistant Isolates	Antibiotic Susceptibility Profile							O-EP	RG
		AMP	GEN	CIP	CHF	TET	CEF	MER		*tetA*			AMP	GEN	CIP	CHF	TET	CEF	MER		*tetA*
S/Sal	*K. oxytoca*	IR	R1	S	S	S	S	S	-		S/Tun	*E. cloacae*	IR	S	S	R1	S	S	S	-	
	*E. hormaechei*	IR	R1	S	S	S	S	S	-			*E. cloacae*	IR	S	S	R1	S	S	S	-	
	*K. pneumoniae*	IR	R1	S	S	S	S	S	-			*E. cloacae*	IR	S	S	R2	S	S	S	+	-
	*E. hormaechei*	IR	R1	S	S	S	S	S	-			*K. oxytoca*	IR	S	S	R1	S	S	S	-	
	*E. cloacae*	IR	R1	S	S	S	S	S	-			*E. kobei*	IR	S	S	R1	S	S	S	-	
	*K. pneumoniae*	IR	R1	S	S	S	S	S	-			*S. liquefaciens*	IR	S	S	R1	S	S	S	-	
	*E. cloacae*	IR	R1	S	S	S	S	S	-			*S. liquefaciens*	IR	S	S	R1	S	S	S	-	
	*K. cryocrescens*	IR	S	S	S	R2	S	S	+	+		*K. oxytoca*	IR	S	S	R1	S	S	S	-	
	*K. cryocrescens*	IR	S	S	S	R2	S	S	+	+		*E. asburiae*	IR	R1	S	S	S	S	S	-	
	*K. cryocrescens*	IR	S	S	S	R2	S	S	+	+		*E. kobei*	IR	R1	S	S	S	S	S	-	
	*K. cryocrescens*	IR	S	S	S	R2	S	S	+	+		*E. asburiae*	IR	R1	S	S	S	S	S	-	
	*K. oxytoca*	IR	S	S	R1	S	S	S	-			*K. pneumoniae*	IR	R1	S	S	S	S	S	-	
S/Tun	*K. oxytoca*	IR	R1	S	S	S	S	S	-		S/JE	*K. pneumoniae*	IR	S	S	R1	S	S	S	-	
	*K. oxytoca*	IR	R1	S	S	S	S	S	-			*E. cloacae*	IR	R1	S	S	S	S	S	-	
	*K. oxytoca*	IR	R1	S	S	S	S	S	-			*E. cloacae*	IR	R1	S	S	S	S	S	-	
	*K. oxytoca*	IR	R1	S	S	S	S	S	-			*E. cloacae*	IR	R1	S	S	S	S	S	-	
	*K. pneumoniae*	IR	R1	S	S	S	S	S	-		S/Shr	*K. pneumoniae*	IR	R1	S	S	S	S	S	-	
	*K. oxytoca*	IR	R1	S	S	S	S	S	-		CF/Shr	*E. cloacae*	IR	S	S	R1	S	S	S	-	
	*K. oxytoca*	IR	R1	S	S	S	S	S	-			*E. cloacae*	IR	S	S	R1	S	S	S	-	
	*K. pneumoniae*	IR	R1	S	R1	S	S	S	-			*E. cloacae*	IR	S	S	R1	S	S	S	-	
	*R. ornithinolytica*	IR	R1	S	S	S	S	S	-												
S/Tun	*R. planticola*	IR	R1	S	S	S	S	S	-												
	*R. ornithinolytica*	IR	R1	S	S	S	S	S	-												
CF/Hal	*E. cloacae*	IR	S	S	R1	S	S	S	+	-											
	*E. cloacae*	IR	S	S	R1	S	S	S	-												
	*E. cloacae*	IR	S	S	R1	S	S	S	-												
	*E. coli*	R	S	S	S	S	S	S	-												
	*E. coli*	R	S	S	S	S	S	S	-												

O-EP—overproduction of efflux pumps; RG—resistance genes; S—sushi, CF—cooled fish; Sal—salmon, Tun—tuna, Shr—shrimps, JE—Japanese eel, Hal—halibut; IR—intrinsic resistance; AMP—ampicillin, CIP—ciprofloxacin, GEN—gentamicin, CHF—chloramphenicol, TET—tetracycline, CEF—ceftazidime, MER—meropenem; S—susceptible, R1—concentration of ATBs according to the European Committee on Antimicrobial Susceptibility Testing, R2—concentration of ATBs according to the Clinical & Laboratory Standards Institute; + positively detected, - not detected.

## Data Availability

Data of the current study are available from the corresponding author on reasonable request.
